# The Healing Effect of Curcumin on Burn Wounds in Rat

**Published:** 2015-01

**Authors:** Davood Mehrabani, Mojtaba Farjam, Bita Geramizadeh, Nader Tanideh, Masood Amini, Mohammad Reza Panjehshahin

**Affiliations:** 1 Stem Cell and Transgenic Technology Research Center, Shiraz University of Medical Sciences, Shiraz, Iran;; 2 Department of Medical Pharmacology, School of Medicine, Fasa University of Medical Sciences, Fasa, Iran;; 3 Organ Transplant Research Center, Shiraz University of Medical Sciences, Shiraz, Iran;; 4 Department of Pharmacology, School of Medicine, Shiraz University of Medical Sciences, Shiraz, Iran;; 5 Laparascopy Research Center, Department of Surgery, Shiraz University of Medical Sciences, Shiraz, Iran

**Keywords:** Burn, Curcumin, Wound, Healing, Rat

## Abstract

**BACKGROUND:**

Burns are still considered one of the most devastating conditions in emergency medicine affecting both genders and all age groups in developed and developing countries, resulting into physical and psychological scars and cause chronic disabilities. This study was performed to determine the healing effect of curcumin on burn wounds in rat.

**METHODS:**

Seventy female Sprague-Dawley 180-220 g rats were randomly divided into 5 equal groups. Groups of A-C received 0.1, 0.5 and 2% curcumin respectively and Group D, silver sulfadiazine ointment. Group E was considered as control group and received eucerin. After 7, 14 and 21 days of therapy, the animals were sacrificed and burn areas were macroscopically examined and histologically were scored.

**RESULTS:**

Administration of curcumin resulted into a decrease in size of the burn wounds and a reduction in inflammation after 14^th^ days. Reepithelialization was prominent in groups A-C while more distinguishable in group C. In group C, epidermis exhibited well structured layers without any crusting. There were spindle shaped fibroblasts in fascicular pattern, oriented parallel to the epithelial surface with eosinophilic collagen matrix.

**CONCLUSION:**

Curcumin as an available and inexpensive herbal was shown be a suitable substitute in healing of burn wounds especially when 2% concentration was applied.

## INTRODUCTION

Burns are still considered one of the most devastating conditions in emergency medicine affecting both genders and all age groups in developed and developing countries, resulting into physical and psychological scars and cause chronic disabilities.^1^ Burn injuries during pregnancy are still important due to the increasing trend in mortality and morbidity of both mother and infant.^2^ Multiple factors affect these morbidity and mortality including the depth and size of the burn. Also, inhalation injury and development of other significant secondary complications also influence the outcome.^3^ Due to the role of flame fire and undertaken suicides, practical activities such as education to decrease this awful event and its physical and emotional complications from responsible governors still seem mandatory.^2^

For survivors, the most persisting problem is scarring, so the process of wound healing and the final outcome of this process is under investigation with the hope of decreasing the problems related to scar. Burn wound healing is a complex process including inflammation, granulation, and remodeling of the tissue.^4^

Many factors contribute to delay the wound repair process such as oxygen free radicals.^5^ Oxidative stress plays an important role to delay healing and contributes to secondary tissue damage.^5^ It is believed that, in burn wound care, early antioxidant therapy, reinforces cellular antioxidant defense mechanisms, decreases free oxygen radicals mediated delay in healing and promotes the healing process of the wound.^5^ Advances in knowledge of wound healing process and improvements in technology have driven the control of infections and antibiotic resistances.^6^

Silver sulfadiazine has been introduced as the gold standard in topical burn therapy and has also antibacterial properties.^7^ Some authors reported it to delay the wound-healing process^8^ and some serious cytotoxic activities on the host cells.^9^ Its silver constituent was shown to be highly toxic to both keratinocytes and fibroblasts.^10^ It may also result into transient leukopenia secondary to bone marrow suppression.^11^ Its conventional anti-infective property which was already demonstrated, can not provide the moisture to promote a rapid wound healing.^11^ Many reports are also available on the resistance of several bacteria to silver sulfadiazine.^6^ Therefore, there is a need for new agents for treatment of burn wounds in health care practice^4^ with less adverse problems and better efficacy.^12^

Traditional medicine was shown to be the treatment of choice in burn wound repair. It was reported that honey has almost equal or even superior effects in burn wound healing when compared with conventional treatments.^13^ Using a traditional Chinese medicine, *Fufang Xuelian* Burn Ointment (FXBO) to treat superficial and deep second-degree burn wounds, it was demonstrated that it was well tolerated and was more effective than control group in treatment of superficial and deep second-degree burn wounds.^14^ Green tea extract (*Camellia sinensis*) was shown to have wound healing properties especially effective on burn wounds healing.^15^

For centuries, the medicinal plants have been extensively used in wound healing of burned injuries.^16^^-^^19^ Among natural antioxidants, curcumin is one of the most potent anti-inflammatory antioxidant in clinical medicine.^20^ Turmeric derived from the plant *Curcuma longa* is a gold-colored indian spice used not only for health care but also for the food preservation.^21^ The diferuloyl-methane part of curcumin has antioxidant and anti-inflammatory properties without toxicity even at high doses.^20^ Curcumin has direct antioxidant properties mediated by regulation of some enzymes, transcription and growth factors, inflammatory cytokines.^22^ This study was performed to determine the healing effect of topical curcumin in burn wounds in comparison to silver sulfadiazine ointment in rat as an animal model.

## MATERIALS AND METHODS

During summer and fall of 2012, in an experimental trial, 70 female Sprague-Dawley 180-220 g rats were randomly divided into 5 equal groups, assigned A to E. Animal selection, all experiments, subsequent care and the sacrifice procedure were all adhered to identical guidelines under supervision of Animal Care Committee of Iran Veterinary Organization. All experiments were carried out under aseptic conditions in Laboratory Animal Center of Shiraz University of Medical Sciences. During the experiments, the animals were housed one per cage, maintained under controlled environmental conditions (21±2ºC, 65–70% RH and a balanced diet with free access to food and water).

Each animal in groups A, B and C received 0.1%, 0.5 and 2% curcumin respectively and in group D, silver sulfadiazine ointment (complete cover of the injured area)*. *Group E animals were considered as controls and were treated with eucerin. To produce the tpica medicatin, the active ingredient, curcumin (Across, USA*) *was used with eucerin as base and tween 20 as solvent to prepare a novel formulation for topical ointment of curcumin, at 3 concentrations of 0.1%, 0.5% and 2%. Silver sulfadiazine (SSD) was provided from Shafa Co, Tehran, Iran**.**

A standard 3rd degree burn wound was produced using a hot plate with the same size about 20% total body surface area (TBSA) and at identical temperature as described before.^12^ The rats were sedated by intramuscular injection of ketamine (15 mg/kg) and xylocain (1.1 mg/kg) and their back hairs were shaved and the skin was cleansed with povidone iodine solution and then wiped with sterile water before induction of experimental burn injuries. 

The wounds were examined every 24 hours and any necrotic tissue was removed. Medications were applied instantly and repeated twice daily. Five rats were excluded from the study due to infected wounds (n=2) and un-standard established wounds (n=3). Five rats also died during the experiment. 

Wounds were daily examined for any changes in appearance of wounds, the color, smell of any discharge and time of scar separation. Animal activity was also recorded if they became lethargic. Pictures were taken by digital camera provided on days 1, 14 and 21 after starting of the experiment and were evaluated by stereology software in the sttereology research center of department of anatomy in Shiraz Medical School which offered us an indirect tool to mesure the burn wound area of each rat. 

**Fig. 1:**
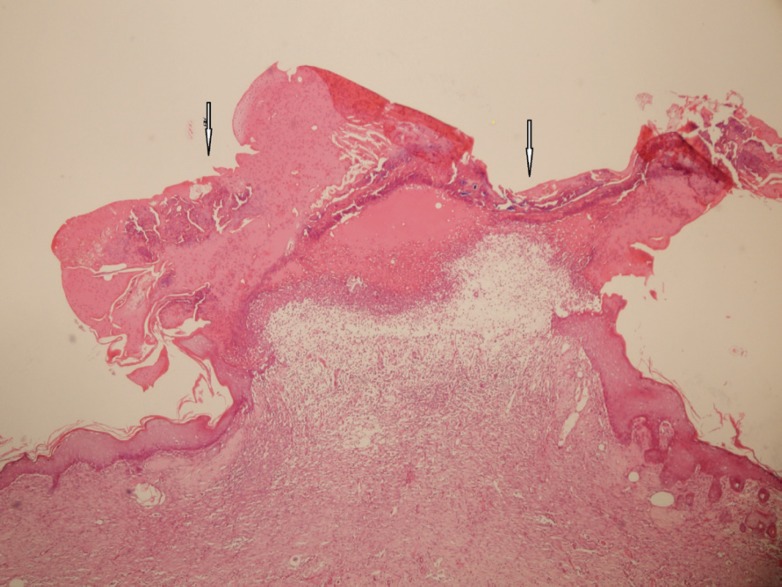
Epidermal ulceration with severe acute inflammation

**Fig. 2:**
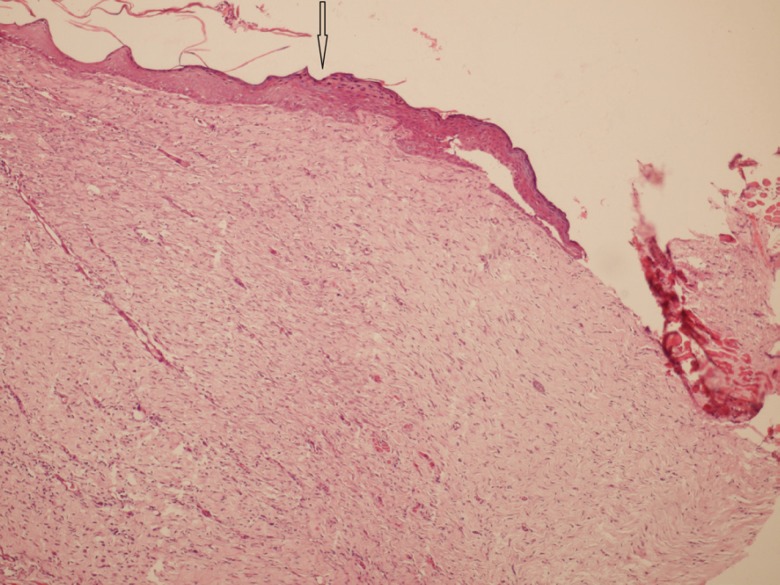
Beginning of re-epithelialization with dermal fibrosis and collagenization

**Fig. 3:**
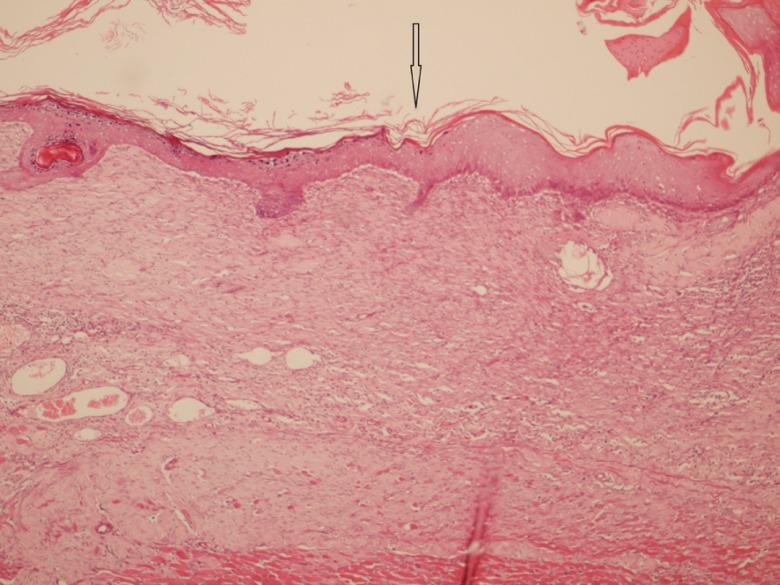
Complete epidermal epithelialization with dermal fibrosis

After 7, 14 and 21 days of treatment, some animals were sacrificed after taking photoes. Sacrification was done with an overdose of anesthetics and the burn areas were removed for histological studies. Hematoxylin and eosin stainings was provided for presence of any reepithelialization, crusting blistering, spongiosis, granulation tissue and collagen matrix organization, inflammation, congestion, edema, etc (Figures 1-3). 

The data were analyzed using SPSS software (version 11.5, Chicago, IL, USA) by non-parametric tests of Kruskal-Wallis and Mann-Whitney with bonferroni correction for multiple comparisons. A p<0.05 was considered statistically significant for Kruskal-Wallis and corrected for Mann-Whitney. 

## RESULTS

A decrease in wound size at days 7 and 14 was seen in all groups, but there was no significant difference among groups regarding the wound areas. Wound areas continued to decrease till the last day of experiment (day 21). Interpretation of photoes by stereology revealed a more prominent wound closure in group C, compared to groups D (*p*=0.005) and E (*p*=0.003). Among curcumin-treated groups the area of wounds were not statistically different. 

In histopathological study regarding the inflammation, there was a decreasing process after days 14 and 21 without any significant difference between groups on day 7 and 14 but on day 21, the difference was statistically significant between curcumin-treated groups (A-C) and group D (*p*=0.001). When compared to group E, the best result was visible in group C (*p*=0.002 for group A; *p*=0.001 for group B; *p*=0.0008 for group C; and *p*=0.003 for group D). 

In the 21th day, reepithelialization was more prominent in groups A-C in comparison to group D while among curcumin-treated groups reepithelialization was more prominent in group C (*p*=0.002 for group A, *p*=0.001 for group B, *p*=0.0001 for group C, and *p*=0.04 for group D). By correction coefficient of *p* value calculated by Bonferroni’s law, group D appears not to have significant difference with non-trated group E regarding reepithelialisation. The epidermis exhibited well structured layers with no crusting. There were spindle shaped fibroblasts in fascicular pattern, oriented parallel to the epithelial surface with eosinophilic collagen matrix. 

In the last day of experiment, granulation tissue formation was not statistically different among groups. In the control group, there was a loose collagen matrix with round to polygonal fibroblasts along with interstitial edema and hemorrhage. There was also infiltration of polymorphonuclear and lymphocytes as well as occupation of dermal layers by adipose tissue. Few well-formed congested capillaries were present within granulation tissue. Incomplete monolayer of epidermal cells with evidence of crusting was also observed. 

## DISCUSSION

Burn as a multifactorial trauma involves pathophysiologic processes of all body organs impairing a person’s psychological, social and physical functioning, esthetic appearance, interpersonal relationships and all aspects of Health Related Quality of Life.^23^ Wound healing and tissue repair in burn injuries are considered as a complex process including inflammation, granulation and remodeling of the tissue. Oxidative stress plays an important role in delay of healing and contributes into poor outcomes. It may be decreased by antioxidant therapy which does not have any clinical implication till now.^5^ The relationship between the amount of produced oxidative radicals and free radicals determines the outcome of local wound repair and systemic damage.^5^ Treatment with antioxidants reinforces cellular antioxidant defense mechanisms, might decrease free radical mediated damage and minimize tissue destruction during burn injury and can accelerate the process of wound healing. ^5^

The *in vivo* wound healing potential of pomegranate extract has been evaluated in rat dermal wound model while a promising phytopharmaceutical healing effect in wounds was noticed due to its major antioxidant constituent, ellagic acid (EA, 13 %, w/w).^24^ *Fagonia indica* Burm f. (Mushikka or white spine) as a folk medicinal plant found in deserts of Asia and Africa was shown to enhance the skin wound re-epithelialization and speed up the healing process in comparison to conventional conventional products.^25^

Curcumin (difeurloyl-methane) is among one of the most potent natural anti-inflammatory antioxidant materials. Curcumin was shown to ameliorate the inflammation in liver diseaseand also to enhance the wound repair when used orally after burn in animal models.^26^^-^^28^ The oral application of curcumin is limited by its relatively low *in vivo * bioavailability. So its topical form may be an ideal alternative for wound healing.^28^ Recent encouraging findings showed that curcumin was a natural pharmacotherapeutic in control of both severe burn pain and in improvement of wound healing.^29^ Kulac *et al.* demonstrated that there was a rise in the expression of proliferating cell nuclear antigen in skin tissues of curcumin treated rat models suffering from burn lesions substantiating the beneficial effects of the topical application of curcumin in acceleration of wound healing.^21^

In this study, we evaluated the *in vivo *properties of topical curcumin on burn wound healing in rats. Reepithelialization and increased migration of myofibroblasts, fibroblasts, and macrophages were more prominent in curcumin -treated groups showing that curcumin played a prominent role in wound healing process after burn injury. The diferuloyl-methane structure of curcumin is responsible for its antioxidant and anti-inflammatory effects and as it lacks toxicity even at high doses, it is an attractive product to be used in healing of burn injuries.^30^

Many of properties of curcumin are through the regulation of various transcription and growth factors, inflammatory cytokines, protein kinases, and several enzymes.^31^ Curcumin protects skin by quenching free radicals and reduces inflammation through inhibition of nuclear factor-B. It accelerates wound healing, improves collagen deposition and increases fibroblast and vascular density in wounds enhancing. It induces transforming growth factor-beta and stimulates angiogenesis and accumulation of extracellular matrix which continues through the remodeling phase of wound repair.^32^

Silver sulfadiazine ointment is a topical antibacterial ointment which is widely applied in prevention and treatment of burn wounds.^33^ It was demonstrated that silver-based dressings have cytotoxic properties and are not recommended unless wound infection is a significant risk.^34^ In 66% of wounds treated with hydrosome gel, healing was faster (1.5-2 days) in comparison with sulfadiazine ointment (9.9±4.5 days *vs.* 11.3±4.9 days, *P*=0.015).^35^

An increase in expression of proliferating cell nuclear antigen in skin tissues of curcumin-treated rats in the burn was reported. The findings clearly substantiate the useful effects of topical use of curcumin in acceleration of wound healing.^21^ Pretreatment with oral curcumin in rats and then by once-daily oral treatment for three days declined the percentage of unburned skin interspaces which progressed to full necrosis.^29^

In our study, reepithelizations was significantly more prominent on day 21 in curcumin groups (significantly in 2% curcumi*n * group) when compared to other groups. It seems that wound healing in control group was not related to euserin and it was just a normal response to wound injury. It can be concluded that topically applied curcumin may be a suitable substitute for silver sulfadiazine especially when 2% concentration is used as a non-toxic, inexpensive and easy to produce product. Further studies should address these issues in humans.

## Note

Please cite this paper as:

Mehrabani D, Farjam M, Geramizadeh B, Tanideh N, Amini M, Panjehshahin MR. The Healing Effect of Curcumin on Burn Wounds in Rat. World J Plast Surg 2015;4(1):29-35.
